# Meteorology as a Collateral Science to the Study of Medicine

**Published:** 1858-02

**Authors:** E. M. Pendleton

**Affiliations:** Sparta, Ga.


ATLANTA

Medical and Surgical Journal.

VOL. III.] FEBRUARY, 1858. [No. VI.

### ORIGINAL COMMUNICATIONS.

### ARTICLE 1.

*Meteorology as a collateral Science to the study of Medicine.*—By E. M. Pendleton, M. D., Sparta, Ga.

Until within the last half century, the science of meteorology had made but little progress, and developed but few facts of much interest to the philosopher. More recently the attention of the scientific world has been directed to this interesting field of human knowledge, with the best results, especially with reference to its connection with climatology and agriculture. Strange that up to the present time nothing has been said or written as to its connection with Hygiene and the etiology of disease. Our own knowledge of meteorology, (having been engaged in taking observations for the Smithsonian Institution for several years,) has led us to inter, that most idiopathic diseases, and many considered as symptomatic, result, from the variable conditions of oxygen, caloric, water and electricity in the atmosphere. Any means that might be instituted to ascertain the exact quantity of these four great principles at given periods and localities, in connection with statistical records of the prevailing diseases, would in our opinion, establish inductively important truths in relation to the etiology of disease, that are totally unknown, or exist as mere theories.

The primary fields of observation, occupied by the meteorologist, may be briefly stated as follows: First, barometrical, or that which estimates the pressure of the atmosphere, and by consequence enables the scientific observer to determine its density at any given period of time, and whether an excess or deficiency of oxygen prevails. Second, the thermometrical, or that which ascertains the thermal condition of the atmosphere, estimates the relative amount of caloric in a cubic foot of air, &c. Third, the hygrometrical, which tells with mathematical precision, the amount of aqueous vapor existing in the atmosphere at the time of taking the observation. Fourth, the electrometrical, which designates the quantity or intensity of the electric fluid in the air at any given time or place.

In connection with these great primary principles, are others not so specially connected, it may be with the elimination of disease, as those above mentioned, such are the fall of rain, snow, hail, dew, &c. ; the force, velocity and course of the wind; the character and extent of the clouds. But, even these to the constant observer, will afford much matter for reflection, and reveal facts in connection with the etiology of disease, which otherwise would remain undeveloped. Thus the force and velocity of the wind might affect the human constitution, either by purifying the atmosphere, by dispersing noxious vapors, or producing sudden changes of temperature, which is, doubtless, a frequent cause of catarrhal fevers, pneumonias, influenzas, and bronchial affections. It not unfrequently occurs, also, that the course of the wind has something to do with the prevalence of epidemics in certain countries, as in England, which has resulted in the quaint couplet,

"The wind that bloweth from the East,
Is good for neither man or beast."

But whatever might be the effect of these irregular meteoro logical phenomena, and, however difficult to arrive at correc- conclusions in reference to them, we doubt not, those primary principles already mentioned, existing as they do, in constant, but variable quantities in the atmosphere, constitute the hidden arcama of disease—the Pandora's box, from which emanates most of the physical ills of human nature.

Oxygen is as much the principle of life, as it is of combustion. It is the great decarbonizer of the life blood, and generator of the life heat. But it requires a certain amount of oxygen, no more nor less, to produce these results properly, and carry on healthy respiration. Too little will not sufficiently neutralize the carbon of the blood, but suffer this poison to escape into the system, and produce stagnation and death; too much, surcharge the whole nervous as well as arterial system with bright florid blood, and results in a dissolution of the system, more or less rapid, according to the overplus of oxygen respired. Now, there are chnages constantly going on in the atmosphere, by which the amount of oxygen, in a given space is increasedor diminished, according to the rise or fall of the mercury in the Toricellian vacuum. When the barometer ranges high, near the point of 30 inches, the atmosphere is much more dense, and contains a greater amount of oxygen in each cubic foot, than when it ranges but little above 29 inches, as it does sometimes for days together. This variation in the condition of the air, in which "we live and move and have our being," must have much to do with the generation of disease, particularly those affecting the respiratory system. We remember some years since, to have treated a case of cardiac asthma, in which much interest was taken, as our diagnosis had been called in question. When there were no other existing causes to bring a paroxysm, we could, almost, always foretell the time of the attack, by the fall of the barometer. As the respiratory element of the atmosphere was diminished, the difficulty in respiration increased and *vice versa.* In fact it has long been known by physicians, and asthmatic patients themselves, that at the beginning of cloudy, damp weather, they are subject to attacks of the disease. Our own observation has taught us, that the very juncture in which all asthmatics are the worst, is when the barometer is the lowest, and there is the least oxygen in the atmosphere. The same principle has long since been elucidated in the ascent of high mountains, where the barometer falls one inch for every thousand feet, and respiration becomes more and more difficult, until in very high altitudes breathing is almost impossible, on account of the small amount of the same vital principle in the air.

The knowledge of these facts, might tend to institute a prophylaxis in preventing paroxysms of asthma which might relieve that class of patients from a great amount of suffering. When it is known that they need oxygen above all else, they will select for a permanent residence those localities, which have been ascertained by meteorological observation to possess the greatest amount of that principle—the seashore for instance, where the barometer ranges about 30 inches. And so of consumptives, whether in the first or last stage, there being a deficiency in the respiratory function, because of the contraction or obliteration of some of the air cells, it becomes necessary to seek an atmosphere where there is an excess of oxygen to aid this deficiency. And, I doubt not, it is owing to this fact that the seashore, in certain localities, has always been considered more efficacious in lung diseases than midland or mountainous regions, though the virtue has been supposed to exist in the salt air itself. The same remarks will apply to every class of respiratory diseases, especially, where dyspnea is an occasional concomitant.

It were perhaps unnecessary for us to attempt to prove that the degree and intensity of caloric in the atmosphere, has much to do with the etiology of disease. Hence there are diseases peculiar to the summer season, when there is an excess of heat, and others which prevail in the winter when there is a deficiency, and so in reference to cold and warm climates.—But mainly would we hope to be benefited by the study of meteorology in this connection by comparing the vicisitudes of atmospherical heat, as indicated by the range of temperature. Our observations have led us to the conclusion that several epidemics attributed to malaria and considered as endemical in their character are dependent upon sudden changes of temperature. We have no doubt that the continued fevers of the rural districts of the South miscalled Typhoid, are of this class. And it would not surprise us to find that many of the Phlegmasia, involving as they do the internal viscera came under the same general head. The *modus operandi* of such inflammations is easily explained upon this theory. During a warm spell of weather the spine is in a relaxed state, the mouths of the exhalents are open and upon a sudden change of temperature they become contracted, as also the capillary system of blood-vessels, the result is that an overplus of the fluids of the body is thrown upon the internal organs, and those suffer most which are in the weakest state. I doubt not many diseases are generated in this way and many chronic affections greatly aggravated. If then the range of temperature has so much to do with the origin of epidemics, how important it is for the profession to engage more fully in the science of Meteorology in connection with the prevalence of diseases in special localities, by which coincidences may be developed in such numbers as to establish by the most indubitable facts, the causes which operate in the production of disease.

The aqueous and electrical conditions of the atmosphere have we doubt not also much to do in originating certain diseases. Hence the Psychrometer or hygrometer may be called into service by the scientific physician, and the amount of humidity in the atmosphere, together with the force of vapor in inches, and the relative fraction of saturation be noted and compared with the statistics of prevalent diseases. By this process we doubt not that much light might be thrown upon certain morbid affections, particularly those of an endemical character. My impression is that malaria depends to a considerable extent upon the aqueous vapors in the air. A bad air is always a humid air. Hence chemists have never been able by the most minute analyses to detect any poisonous gases floating in the air where endemical diseases prevail. Possibly if they had sought for them in the aqueous particles of the atmosphere instead of the air itself, they might have been detected. Heat, moisture and vegetable putrefaction are supposed to be the generators of malaria. As it is not a gas it must be held in solution by the aqueous vapors of the air. This seems to be proven by the fact that at night when vapors are more prevalent, malaria is more intense. Hence an unacclimated person may pass through a malarious district without harm unless he tarries all night, in that event he is almost certain to take the fever; might it not be possible that well conducted hygrometrical observations in malarious districts, might tend to lift the darkness that envelopes the whole subject of the etiology of endemical diseases. At least if the aqueous theory be true, it would teach the intensity of the poison held in solution by the amount of vapor in the air, and its claims to truth might be vindicated, by the comparative prevalence of malarious fevers during seasons of dryness or humidity as tested by the hygrometer.

In close connection with the humidity of the atmosphere, is its electrical condition, for the vapors which rise from the earth, are always positively electrified, and one of the most abundant sources of atmospherical electricity, is evaporation. By the electrometer the exact electrical state of the atmosphere may be ascertained both as to its character and intensity.—From the fact that this principle pervades all nature and is subject to such rapid variations, that it is intimately connected with animal and vegetable life, that it has to do not only with our physical but our moral and mental being, elevating or depressing the spirits according to the state of the nervous system, or even it may be energizing the process of thought, as acted on by the currents of electricity in the air, the candid investigator after truth is forced to admit that it has much to do with the developement of all that class of diseases in which the nervous system is implicated. Such being the case, how interesting would be the process for investigating the causes of disease by the actual statistical exhibit of their developement, in connection with the phases of electricity in the atmosphere as ascertained by the electrometer.

The limits of this paper will only admit of my introducing the plan, which in my opinion will be productive of the best possible results in the public hygiene, and the advancement of medical science. Already has the Smithsonian Institution made many valuable contributions to Meteorological science by the establishment of various points of observation throughout the country and the world, and we have but very recently received from them papers on the connection of Meteorology with agriculture, climatology &c. So exact have been the calculations made by this powerful calculus, as that isothermal lines have been drawn and maps made indicating the thermal and barometrical conditions of every part of the country. We would simply suggest that the Smithsonian Institution endeavor through their agents, to secure the concurrence of medical practitioners throughout the land to keep statistical records of the diseases prevailing in the localities where Meteorological observations are taken. Thus when a physician is sent for to a patient, let him determine fully the character of the disease and enquire well into its history; when he ascertains the exact day of its inception, let him note down that day, the name of the patient, disease &c. Thus in the prevalence of a particular epidemic of any kind, the conditions of the atmosphere will be ascertained by one class of observers, the cases of disease by another, and much light will necessarily be thrown on their etiology. The medical practitioners in any special locality might combine and have both classes of observations taken for their own benefit in the absence of any general observations of the kind.

We are already in possession of many statistical facts bearing on this subject, which we may hereafter offer to the public more in detail. Our present intention only being to direct the profession, and the Smithsonian Institution particularly, to a new and interesting field of scientific investigation, by drawing an outline of some of the general principles involved in this question. We must desist from prosecuting the subject any farther at present.

