# Erratum

**Published:** 1858-03

**Authors:** 


### Erratum.

Page 324, last line, in February No. article of Dr. Pendleton, for *spine* read *skin*.

